# RovM and CsrA Negatively Regulate Urease Expression in *Yersinia pseudotuberculosis*

**DOI:** 10.3389/fmicb.2018.00348

**Published:** 2018-02-27

**Authors:** Qingyun Dai, Lei Xu, Lu Xiao, Kaixiang Zhu, Yunhong Song, Changfu Li, Lingfang Zhu, Xihui Shen, Yao Wang

**Affiliations:** ^1^State Key Laboratory of Crop Stress Biology for Arid Areas and College of Life Sciences, Northwest A&F University, Yangling, China; ^2^College of Life Sciences, Northwest A&F University, Yangling, China; ^3^Shaanxi Key Laboratory of Agricultural and Environmental Microbiology, College of Life Sciences, Northwest A&F University, Yangling, China

**Keywords:** urease activity, *Yersinia pseudotuberculosis*, RovM, carbon storage regulator system A (CsrA), nutrient

## Abstract

Urease acts as an important acid resistance system and virulence factor that is widespread among microorganisms. RovM is a global regulator that regulates a series of genes and pathways including acid survival systems in the enteric bacterium *Yersinia pseudotuberculosis (Yptb)*. However, whether RovM regulates the urease activity in *Yptb* was still unknown. In this study, by using qualitative and quantitative urease assays, we show that the urease expression responds to nutrient conditions and the RovM protein represses urease expression by binding to its promoter. A previously reported positive regulator OmpR activates urease activity but RovM plays a dominant role in different nutrient conditions. In addition, carbon storage regulator system A (CsrA), the upstream regulator of RovM, dramatically down-regulates urease activity possibly by its binding to the Shine-Dalgarno (SD) sequence of the mRNA encoding the urease. In conclusion, this study demonstrates that urease activity is strictly controlled by nutrient conditions and is down-regulated by the CsrA-RovM pathway.

## Introduction

*Yersinia pseudotuberculosis* (*Yptb*) is a Gram-negative enteropathogenic bacterium and main transmitted through the contacting with infected animals and eating contaminated food (Fukushima et al., 1988; Fukushima and Gomyoda, 1991; Laukkanen et al., 2008). Given the fact that food-borne pathogens must withstand stomach acidity, survival in acidic conditions is essential for *Yptb*. To counteract this acidic condition, bacteria have developed sophisticated acid resistance systems (Bearson et al., 1997). Most studies were conducted on *Escherichia coli, Salmonella typhimurium, Helicobacter pylori*, etc., with *E. coli* being the most widely studied (Lin et al., 1995; Pflock et al., 2006; Yu et al., 2011). At least four acid resistance (AR) systems, named AR1 to AR4 are generally recognized, which are known to allow the bacteria survival under extremely acidic conditions (reviewed in Foster, 2004; Zhao and Houry, 2010). Four clusters of Type VI secretion system (T6SS-1 to T6SS-4) have been identified in *Yptb* (Zhang et al., 2011) Recently, the T6SS-4 was reported to play a role in resistance to mild acid stress which was activated by OmpR to maintain cell functions in *Yptb* (Zhang et al., 2013).

The urease system is another important acid resistance system in many enteropathogens, which increases survival under acid stress (Young et al., 1996; Hu et al., 2009). The hydrolysis of urea as a specific substrate in the environment by urease will produce CO_2_ and NH_3_ and thus relieves the acid stress on the bacteria. In the acidic environment, the presence of urea drastically increased the survival rate of the wild type *Yptb* but this was not observed in the urease mutant (Riot et al., 1997). This finding demonstrates that the urease system exerts an acid-resistance function in *Yptb*. The most widely studied urease acid resistance system is that of *Helicobacter pylori*, which lives in the strong acid of its host’s stomach (Marshall et al., 1990). Urease not only provides an easy-to-use nitrogen source for bacteria and relieves acid stress, it is also considered to be a universal virulence factor (Rutherford, 2014).

Acid resistance systems play important roles in the physiology and virulence of enteropathogenic bacterium, thus their expression is strictly regulated by different factors. By using two-component regulon assays, Flamez et al. (2008) have revealed several regulators including PhoP, OmpR, and PmrA control acid survival in *Yptb*. Among those regulators, by positively regulating urease expression, OmpR plays a vital role in increasing the survival rate of bacteria under acid stress (Hu et al., 2009). The transcriptional multi-regulatory, RovM also has been reported to regulate the acid survival systems including AR3- and T6SS4-dependent systems (Song et al., 2015). RovM possesses the classical structure of an HTH (helix-turn-helix) domain at the N-terminus and an inducer binding domain at the C-terminus and it is a global regulatory factor belonging to the LysR family (Quade et al., 2011). RovM is regulated by environmental factors and its expression is generally induced by nutrient restriction (Heroven and Dersch, 2006). In addition, RovM was shown to be regulated by the carbon storage (Csr) system (Heroven et al., 2008).

The Csr system is involved in the regulation of metabolism and fundamental physiological properties (reviewed in Romeo et al., 2013). The Csr system is composed of three members: CsrA, CsrB and CsrC. CsrB and CsrC are two independent non-coding RNAs that sequester the dimerized RNA binding protein, CsrA (Liu et al., 1997; Weilbacher et al., 2003). CsrA plays important roles in regulating carbon metabolism and motility (Romeo, 1998; Wei et al., 2001; Baker et al., 2002; Jackson et al., 2002) biofilm formation (Jackson et al., 2002) and other biological processes. In *E. coli*, CsrA binds to target mRNA to activate or repress gene expression (Baker et al., 2002; Dubey et al., 2003; Wang et al., 2005; Jonas et al., 2008). Specifically, it binds to the Shine-Dalgarno (SD) sequence in mRNA on the conserved sequence “A(N)GGA” (Kulkarni et al., 2014) and blocks the binding of ribosomes, thereby preventing translation and promoting mRNA degradation (Liu et al., 1995; Liu and Romeo, 1997; Baker et al., 2002; Lucchetti-Miganeh et al., 2008). In addition, it is also reported that the *rovM* expression was up-regulated by CsrA (Heroven et al., 2008).

However, whether regulators such as RovM and CsrA could affect urease expression in *Yptb* and the underlying mechanisms are still unknown. Meanwhile, whether the urease acid resistance system in *Yptb* could also react to different nutrient conditions is still unknown. In this study, we first studied the effect of different nutrient conditions on urease activity. We next investigated the function of the global regulator RovM and CsrA in regulating urease expression. We found that urease expression is dependent on nutrient conditions and the urease activity is negatively regulated by RovM and CsrA in *Yptb*.

## Materials and Methods

### Bacteria Strains and Growth Conditions

Bacterial strains and plasmids used in this study are listed in Supplementary Table S1. The *Yersinia pseudotuberculosis* YPIII was the parent of all derivatives used in this study. *Yersinia pseudotuberculosis* YPIII was cultured at 26°C with appropriate antibiotics in YLB (Yersinia Luria-Bertani medium) medium [1% (w/v) tryptone, 0.5% (w/v) yeast extract, 0.5% (w/v) NaCl, pH = 7.0], M9 minimal medium [6 g/L Na_2_HPO_4_, 3 g/L KH_2_PO_4_, 1 g/L NH_4_Cl, 0.5 g/L NaCl, 1 mM MgSO_4_, 0.1 mM CaCl_2_, 0.2% (w/v) glucose) and Bacto Tryptic Soy Broth (TSB) medium. *E. coli* were cultured at 37°C with appropriate antibiotics in Luria-Bertani broth [LB, 1% (w/v) tryptone, 1% (w/v) yeast extract, 0.5% (w/v) NaCl, pH = 7.0]. Antibiotics were added with following concentrations: 20 μg/mL nalidixic acid, 20 μg/mL chloramphenicol, 100 μg/mL kanamycin, 50 μg/mL ampicillin.

### Plasmid Construction

Primers used in this study are listed in Supplementary Table S2. All amplification, restriction digestion, ligation and transformation were performed as standard molecular and genetic techniques (Miller, 1992). The in-frame deletion mutants were constructed as described before (Wang et al., 2015; Lin et al., 2017). To construct the knock-out plasmid for deletion of the Δ*ureC* (Ypk_1133) gene, the 774 bp upstream fragment and the 655 bp downstream fragment flanking *ureC* were amplified with primer pairs Ypk_1133M1F Sal I/Ypk_1133M1R and Ypk_1133M2F/Ypk_1133M2RBgl II. The upstream and downstream PCR fragments were ligated by overlapping PCR and the resulting PCR products were inserted into the Sal I/Bgl II sites of the vector pDM4. The plasmid to generate Δ*rovM* mutant was constructed in a similar manner by using primers listed in Supplementary Table S2. The plasmid to generate Δ*ompR* mutant was constructed in the previous study by our group (Zhang et al., 2013). To construct the complementation plasmid pKT100-*rovM*, primers rovMcom-F-BamH I/rovMcom-R-Sal I were used to amplify *rovM* gene fragment from *Yptb* genome. The PCR product was inserted into pKT100 by using the BamH I/Sal I sites. To construct the overexpression plasmids pKT100-*csrA* and pKT100-*csrA(R44A)*, primers listed in Supplementary Table S2 were used. Site-directed mutagenesis was performed by overlap PCR to substitute the arginine residue at position 44 of Ypk_3372 (CsrA) into an alanine residue [CsrA(R44A)]. Briefly, two rounds of PCR were used to amplify the DNA of mutant CsrA(R44A). Primer pairs csrAexF BamH I/csrA-R44A M1R and csrA-R44A M2F/csrAexR Sal I were used to amplify segments 1 and 2, respectively. The second round of PCR was performed by using csrAexF BamH I/csrAexR Sal I as the primer pair, whereas segment 1 and segment 2 (product from the first round of PCR) together were used as templates to get the CsrA(R44A) fragment. The CsrA(R44A) DNA product was digested by BamH I/Sal I and inserted into similarly digested pKT100 to produce pKT100-*csrA(R44A)*.

The *lacZ* fusion reporter vector pDM4-*P_ureABC_ :: lacZ* was made by transcriptional fusion of the urease promoter (*ureABC*, amplified with a primer pair ureABCp1000F-Sal I/ureABCpR-Xbal I from *Yptb* genomic DNA) to the *lacZ* reporter gene. The PCR product was digested with Sal I/Bgl II and inserted into suicide pDM4 vector to produce pDM4*-P_ureABC_ :: lacZ* by using the Sal I/Bgl II site. To express His6-tagged RovM, CsrA and CsrA(R44A), primers rovMF-BamH I/rovMR-Sal I, csrAF-BamH I/csrAR-Sal I and csrAexF BamH I/csrAexR Sal I were used to amplify *rovM, csrA* and *csrA(R44A)* fragments. The PCR products of *rovM, csrA* and *csrA(R44A)* were inserted into the pET28a plasmid to generate pET28a-*rovM*, pET28a-*csrA* and pET28a-*csrA (R44A)* constructs using the BamH I/Sal I sites.

### Urease Qualitative Assays

Phenol red was used as an indicator of the pH changing caused by urease hydrolysis. Qualitative urease tests were performed as described previously (Young et al., 1996). Bacteria were cultured at 26°C and were collected in late-exponential phase. The pellet was washed by PBS buffer for twice and was resuspended in 2 mL qualitative test buffer [0.5% (w/v) NaCl, 0.2% (w/v) KH_2_PO4, 0.2% (w/v) urea, 0.002% (w/v) phenol red]. Then the medium was shaken for 4 h at 26°C and the color of the medium indicates urease activity. The urea is hydrolyzed to ammonia by the urease in the bacteria, causing an increase in the pH of the buffer and this will be detected by a change in color of the buffer (Young et al., 1996).

### Urease Quantitative Assays

Urease activity was quantitated by determining the rate of ammonia produced from the hydrolysis of urea (Young et al., 1996). Briefly, bacteria were cultured at 26°C and were collected in late-exponential phase. The pellet was washed by PBS buffer for twice and resuspended. Five microliters of bacterial suspension was added into 40 μL test buffer [0.1% (w/v) cetyldimethylammonium bromide (CTAB), 0.6% (w/v) NaCl, 100 mM citrate, 5 mM urea, pH = 6.0] and mixed and incubated with shaking. The reaction was terminated by adding 100 μL phenol nitroprusside and then 100 μL alkaline hypochlorite. After 30 min at ambient temperature, absorbance at 635 nm was measured. The protein concentration was quantified by Bradford method, with calibration plot using BSA as standard. Ammonia concentrations were determined by constructing a standard curve using defined concentrations of NH_4_Cl prepared fresh in the same buffer used for the assay (Young et al., 1996). Urease activity was expressed as micromoles of ammonia produced per minute per milligram of protein.

### β-Galactosidase Assays

A transcriptional *lacZ* fusion reporter pDM4*-P_ureABC_ :: lacZ* vector was constructed and transformed into *E. coli S17-1 λ-pir*, then conjugated with *Yptb* (Simon et al., 1983; Milton et al., 1996). The transconjugants were selected on LB agar medium containing chloramphenicol. Target fusion strains were cultured to stationary phase at 26°C with appropriate pH in YLB or M9 medium. β-galactosidase activity was measured according to the Miller method (Miller, 1992), by using ONPG (*o*-Nitrophenyl-β-D-galactopyranoside) and the results were calculated as following [1000^∗^(OD_420_ - 1.75^∗^OD_550_)/(vol (mL)^∗^*t* (min)^∗^ OD_600_].

### Acid Survival Assays

*Yptb* was cultured in M9 medium to mid-exponential phase at 26°C, bacteria were harvested and washed twice with PBS buffer. Next, the pellet was resuspended and diluted 100-fold into pH = 4.2 EG buffer (73 mM K_2_HPO_4_, 17 mM NaNH_4_HPO_4_, 0.8 mM MgSO_4_, 10 mM citrate and 0.4% glucose) with or without 5 mM urea, shaking cultured at 26°C for 30 min. After acid stress, the cultures were serially diluted and plated onto YLB agar plates. After 24 h growth at 30°C, colonies were counted and the survival rate was calculated by dividing the colony forming unit (CFU) number of the stressed cells by the CFU number of the untreated cells.

### Quantitative Real-Time PCR (qRT-PCR)

Bacteria were harvested at the mid-exponential phase and RNA was extracted using RNAprep Pure Cell/Bacteria Kit (TIANGEN, Beijing, China), major steps refer to the protocol. The purity of RNA was determined by agarose gel electrophoresis and the concentration was determined by NanoDrop 2000C (Thermo). The first-strand cDNA was reversely transcribed by using *TransScript First-strand cDNA SuperMix* (TransGen Biotech) with 500 ng RNA. Quantitative real-time PCR (qRT-PCR) was performed by using *TransStart Green qPCR SuperMix* (TransGen Biotech) in CFX96 Real-Time PCR Detection System (Bio-Rad) with SYBR/FAM mode only. The parameters applied in the PCR procedure as follow: 95°C 30 s, (95°C, 15 s; 50°C, 30 s) × 40 cycles. All primers are listed in Supplementary Table S2. The relative expression levels of the target genes were normalized to that of the housekeeping gene 16S rRNA. Final gene expression was calculated by using 2^-ΔΔCT^ method.

### Protein Expression and Purification

His-tagged proteins were used in this study, plasmid pET28a-*rovM*, pET28a-*csrA* and pET28a-*csrA (R44A)* was transformed into *E. coli* TransB (DE3). Bacteria were cultured in LB medium at 37°C to OD_600_ = 0.4, then added 0.5 mM isopropyl β-D-thiogalactoside (IPTG) to induce protein expression for 12 h at 26°C. Then the cells were harvested and lysated by sonication. His⋅Bind Ni-NTA resin (Novagen) was employed to purify the protein following the manufacturer’s instructions. The concentration of purified proteins was measured by Bradford method. The purity of the recombinant proteins was confirmed by SDS-PAGE (sodium dodecyl sulfate-polyacrylamide gel electrophoresis).

### Electrophoretic Mobility Shift Assays (EMSA)

The EMSA was performed as previous (Zhang et al., 2013) with minor modifications. Briefly, the probe (187 bp) of *ureA* promoter was amplified by primer set ureABC-F/ureABC-R, and then the product was retrieved in SDS-PAGE and the concentration was determined. LightShift^®^ Chemiluminescent EMSA Kit (Thermo Scientific) was used following manufacturer’s instructions. Briefly, 20 nM DNA probe was incubated with increasing concentrations (0.3, 0.6, or 1.2 mg/mL) of His-RovM in EMSA buffer (2 μL 2 × binding buffer, 1 μL NP-40, 1 μL 100mM MgCl_2_, 1 μL 20 mM EDTA, 1 μL 50% glycerol, for 20 μL system) for 20 min at room temperature. The electrophoresis performed in 6% native polyacrylamide gel in 0.5 × TBE (Tris-borate-EDTA) containing 5% glycerol electrophoresis buffer for 2 h at 4°C. And the DNA probe was detected using SYBR Green.

### DNase I Footprinting Assays

Footprinting assays were performed as previously reported (Wang et al., 2012) with minor modifications. In short, the *ureA* promoter was amplified with primers PureABC-F/PureABC-R. Then the target fragment was cloned into the pEASY-T1 vector (TransGene) which was further used as a template for the preparation of fluorescent FAM-labeled probes with primers M13R (FAM labeled) and M13F. The FAM-labeled probes were purified using Wizard SV Gel and PCR Clean-Up system (Promega) and were quantified with NanoDrop 2000C (Thermo). To perform the DNase I footprinting assay, different amounts of His_6_-RovM were incubated with 400 ng probes in a total volume of 40 μL in the same buffer. After incubation for 30 min at 30°C, 10 μl solution containing 0.010 U DNase I (Promega), and 100 nM freshly prepared CaCl_2_ was added in 10 μL solution and further incubated for 1 min at 25°C. The reaction was terminated by adding 140 μL DNase I stop solution (200 mM unbuffered sodium acetate, 30 mM ethylenediamine tetraacetic acid and 0.15% SDS). Next, samples were extracted by using phenol/chloroform and were precipitated with ethanol. Then the pellets were dissolved in 35 μL double-distilled water (ddH_2_O). The GeneScan-LIZ500 size standard (Applied Biosystems) was employed. The DNA ladder preparation, electrophoresis and data analysis were performed as described previously (Wang et al., 2012).

### Analytical Size Exclusion Chromatography

Analytical size exclusion chromatography (SEC) was used to monitor binding between protein and target RNA sequences. Herein, SEC was used to detect whether CsrA protein could bind to a synthetic RNA. The sequences of synthetic RNA oligonucleotides were derived from the SD sequence of the mRNA encoding the urease and contained the predicted CsrA binding sites (5′-CUUUCACUUUCUUAACAUGAUACAGGAGGGCUUAUG-3′, 36 bp). The assay was performed according to the previous study (Kulkarni et al., 2014). Briefly, a Superdex 75HR 10/30 analytical column (GE Life Sciences) was calibrated using Gel Filtration LMW Calibration Kit (GE Life Sciences). It contained aprotinin (6.5 kDa)/ribonuclease A (13.7 kDa), carbonic anhydrase (29 kDa), ovalbumin (43 kDa), conalbumin (75 kDa), and blue dextran 2000. The SEC assay was performed as follows: (i) The Superdex 75HR 10/30 analytical column was washed with two volumes of ultrasonically degassed phosphate buffer (50 mM NaCl, 25 mM potassium phosphate buffer, pH = 7.0), the absorbance at 280 nm of the eluate was monitored until the level was stable. (ii) 50 μM CsrA protein and 25 μM synthetic RNA was mixed in the phosphate buffer and incubated with on ice for 1 min. Next, the RNA-protein mixture was loaded to the column carefully. (iii) Started the measurement step, the flow rate was set to 0.5 mL/min and the absorbance at 280 nm of the elution was monitored. (iv) When the measurement of each sample was completed, the column was washed thoroughly and the data was saved and collected. All liquid used in this assay was ultrasonically treated to remove the gas in the fluids.

### mRNA Half-Life Assays

mRNA half-life assays were performed as described (Yakhnin et al., 2013) with minor modifications. *Yptb* was cultured in M9 at 26°C to late-stationary phase. Rifampicin (200 mg/mL) was added to prevent transcription initiation. Aliquots were removed as 1 mL at series times and added to an equal volume of frozen buffer (10 mM Tris-HCl, pH 7.2, 5 mM MgCl_2_, 25 mM sodium azide, 12.5% ethanol and 500 mg/mL chloramphenicol). Bacteria RNA were extracted by using RNAprep Pure Cell/Bacteria Kit (TIANGEN, Beijing, China). The purity of RNA was confirmed by agarose gel electrophoresis. qRT-PCR was performed as described above.

### Statistical Analysis

All results are presented as averages and SDs (standard deviations), the Student’s unpaired *t*-test was used to characterize the difference. The difference of statistically significant is set at *P* < 0.05. GraphPad Prism 5.0 Software was used in concrete.

## Results

### Urease Expression Depends on Nutrient Conditions

**FIGURE 1:**
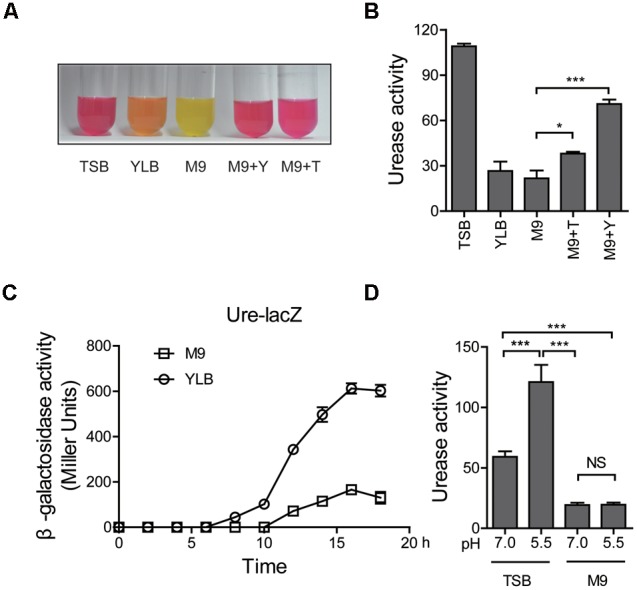
Urease expression depends on nutrient conditions. **(A)** Qualitative assays of urease activity for *Yersinia pseudotuberculosis* (*Yptb*) wild type (WT) grown in medium with various nutrient conditions [TSB, YLB, M9, M9 + Y (yeast extract) and M9 + T (tryptone)]. pH was indicated by phenol red. **(B)** Quantitative assays of urease activity in medium with various nutrient conditions [TSB, YLB, M9, M9 + Y (yeast extract) and M9 + T (tryptone)] for *Yptb* wild type (WT). Urease activity is expressed as micromoles of ammonia produced per minute per milligram of protein. **(C)** β-galactosidase assays of urease promoter activity of the *Yptb* wild type (WT) strains cultured over the entire growth cycle, comparing urease expression in YLB and M9 medium. **(D)** Quantitative assays of for *Yptb* wild type (WT) grown in TSB and M9 medium with various pH conditions (pH = 7.0 or pH = 5.5). Urease activity is expressed as micromoles of ammonia produced per minute per milligram of protein. Data shown are the averages and SDs (standard deviations) from at least three independent experiments. ^∗∗∗^*p* < 0.001; ^∗^*p* < 0.05; NS, not significant.

According to previous reports, several acid tolerance systems respond to nutrient conditions (Song et al., 2015). As urease acts as an important acid tolerance system, we asked whether it also responds to nutrient signals. This led us to explore the effect of nutrients on the expression of urease system. A series of medium with various nutrient profiles were used to evaluate the urease activity. According to the established urease activity measuring method used by Young et al. (1996), phenol red was used as an indicator to monitor pH change (Supplementary Figure S1). The result showed that urease is highly expressed in the rich-nutrient medium such as TSB (Tryptone Soya Broth) compared to that in the minimal medium such as M9 (**Figure 1A**). Consistently, the quantitative assay showed similar results (**Figure 1B**). The urease activity was significantly up-regulated in minimal medium M9 when it supplied with nutrients such as yeast extract or tryptone (**Figures 1A,B**). To further confirm that the urease expression was affected by nutrient conditions, we generated a transcriptional chromosomal *P_ureABC_ :: lacZ* fusion reporter of which the β-galactosidase activity represents urease promoter activation (Supplementary Figure S2). As shown in **Figure 1C**, during the overall growth stages, the urease promoter activation in TSB medium is higher than that in the M9 medium. All these results indicate that different nutrient levels strongly affect urease expression, but the underlying mechanism is still elusive. Since urease is involved in acid resistant systems, we evaluated urease activity in different pH. Consistent with the previous report, urease was activated by low pH in rich-nutrient medium TSB (**Figure 1D**) (Young et al., 1996). Taken together, all these results indicated that urease expression depends on nutrient conditions.

### RovM Negatively Regulates Urease Expression in *Yptb*

**FIGURE 2:**
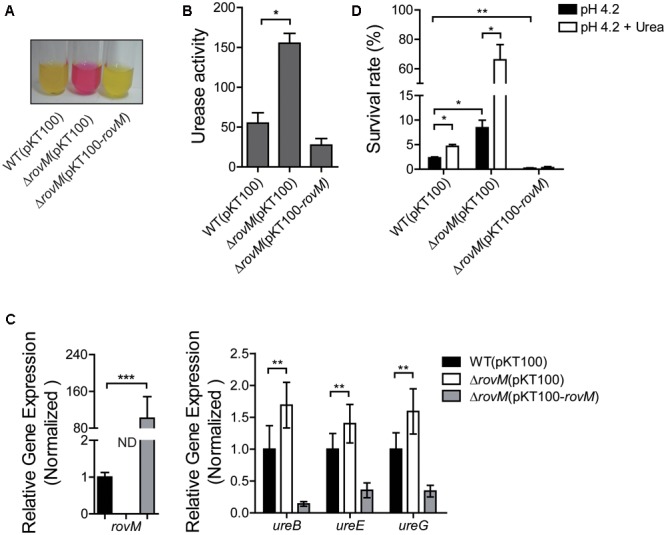
RovM significantly represses urease expression. **(A)** Qualitative assays of urease activity under pH = 4.5 for the *Yersinia Pseudotuberculosis* (*Yptb*) wild type [WT(pKT100)], Δ*rovM* mutant [Δ*rovM*(pKT100)] and *rovM*-complemented [Δ*rovM*(pKT100-*rovM*)] strains. **(B)** Quantitative assays of urease activity for the *Yptb* wild type [WT(pKT100)], Δ*rovM* mutant [ΔrovM(pKT100)] and *rovM*-complemented [Δ*rovM*(pKT100-*rovM*)] strains. Urease activity is expressed as micromoles of ammonia produced per minute per milligram of protein. **(C)** Relative RNA levels of the urease *ureB, ureE, ureG*, and *rovM* genes in *Yptb* wild type [WT(pKT100)], Δ*rovM* [Δ*rovM*(pKT100)] and *rovM*-complemented [Δ*rovM*(pKT100-*rovM*)] strains. The gene expression level was normalized to 16S rRNA. The gene expression of WT(pKT100) was set as 1. **(D)** Survival rates of the *Yptb* wild type [WT(pKT100)], Δ*rovM* [Δ*rovM*(pKT100)], and *rovM*-complemented [Δ*rovM*(pKT100-*rovM*)] strains in pH 4.2 EG buffer with or without 5 mM urea. Data shown are the averages and SDs (standard deviations) from at least three independent experiments. ^∗∗∗^*p* < 0.001; ^∗∗^*p* < 0.01; ^∗^*p* < 0.05; ND, not detected.

RovM is a global regulator that can be activated by nutrient-restricted conditions (Heroven and Dersch, 2006). Furthermore, RovM also has been reported to participate in several acid resistance systems, including the AR3- and T6SS4-dependent acid survival systems (Song et al., 2015). Therefore, to investigate the relation between RovM and urease system, we constructed a *rovM* mutant and measured the urease activity in this mutant. As shown in **Figure 2A**, urease activity was much higher in the Δ*rovM* strain compared to that in the wild type strain, while urease activity was returned to normal level when *rovM* was introduced again [Δ*rovM*(pKT100*-rovM*)]. The quantitative urease activity assay further confirmed this result (**Figure 2B**). We also noticed that urease activity in the complemented strain is even lower than that in the wild type (**Figure 2B**). A possible explanation is that RovM level in the complemented strain is most likely higher than in the wild type, since the pKT100 plasmid is a multi-copy plasmid. As expected, the *rovM* transcription level in the complemented strain was significantly higher than that in wild type (**Figure 2C**). This further confirms that urease activity in *Yptb* was negatively regulated by RovM. Next, the qRT-PCR analysis showed that expression level of three genes (*ureB, ureE*, and *ureG*) in the urease gene operon was significantly increased when *rovM* was deleted. The expression level of these genes in complemented strain was even lower than that in wild type (**Figure 2C**). This further confirmed that RovM negatively regulates urease expression. To further elucidate the role of RovM in acid resistance systems, we examined the survival rate of Δ*rovM* mutant under acid stress. The *rovM* mutant showed a higher survival rate in pH 4.2, while adding the specific substrate urea led to the increase of survival rate in both wild type and Δ*rovM* strains (**Figure 2D**). Similarly, the complemented strain that had higher *rovM* expression showed very low survival in the acid challenge (**Figure 2D**), and this may due to the repressed urease function. Taken together, these results demonstrated that RovM represses urease activity at the transcriptional level.

### RovM Represses Urease Expression by Directly Binding to the Urease Promoter

**FIGURE 3:**
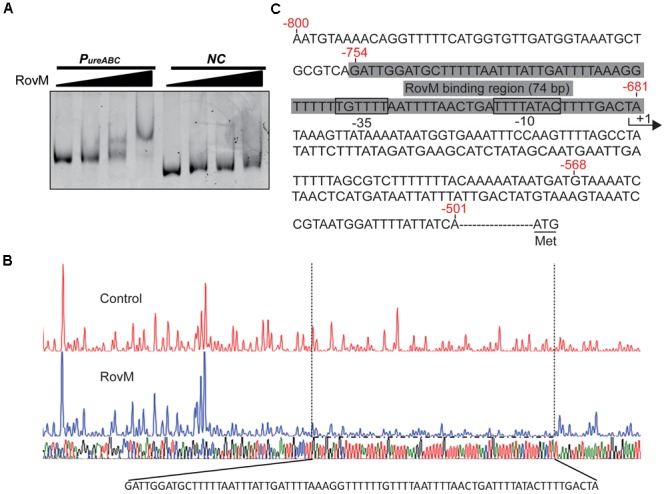
RovM directly binds to the urease promoter. **(A)** Gel retardation assay analysis of the interaction between RovM-His_6_ and the urease promoter (*P_ureABC_*), a fragment derived from the coding sequence (CDS) of urease structural gene *ureC* was used as a negative control. Probe concentrations were 20 ng/μL with increased protein concentration (0.3, 0.6, and 1.2 μM). As a negative control (*NC*), a fragment of *ureC* (urease structural gene) was subjected to the same protein concentration gradient. **(B)** DNase I footprinting assay identified RovM binding sites in the urease promoter region. **(C)** Nucleotide sequences of the urease promoter region [from –800 to –500 relative to the ATG start codon of the first ORF (open reading frame) (Ypk_1131, *ureA*) of the urease operon]. The probe used for EMSA was amplified from –754 to –568 relative to the ATG start codon of the first urease ORF (Ypk_1131, *ureA*). The red region denotes the RovM binding site identified in the DNase I footprinting assay extending from –754 to –681 (74 bp) upstream of the initiation codon of the first urease ORF (*ureA*, Ypk_1131)]. The RovM binding site identified with the DNase I footprinting assay was indicated by shading. Putative –35 and –10 elements of the urease promoter are boxed. +1 denotes the transcription start point. The red number above the nucleotide sequence indicates the position relative to the ATG start codon of the first urease ORF (Ypk_1131, *ureA*).

We have proven that RovM negatively regulates urease activity. However, the underlying mechanism is not clear. Given the fact that RovM is a regulatory protein and it regulates two acid resistance systems by binding to their promoter (Song et al., 2015), we hypothesized that RovM may regulate urease by binding to its promoter. To test this hypothesis, we performed an electrophoretic mobility shift assay (EMSA) to analyze the interaction of RovM with urease promoter. Incubation of RovM-His_6_ with the probe amplified from the urease promoter sequence [–754 to –568 relative to the ATG start codon of the first ORF (open reading frame) (Ypk_1131, *ureA*) of the urease operon] led to the formation of DNA-protein complexes and a gradual shift in band retardation was observed with increased RovM-His_6_ concentration (**Figure 3A**). To further recognize the precise RovM binding site, DNase I footprinting analysis was conducted to identify the DNA region protected from DNase I digestion. A protected DNA region extending from –754 to –681 (74 bp) upstream of the initiation codon of the first urease ORF (Ypk_1131, *ureA*) with high affinity to RovM was identified (**Figure 3B**). By using the online bacterial promoter prediction program BPROM, (**Figure 3C**), both the –10 and –35 regions of the urease promoter were identified within these binding sequences. In summary, these results suggested that RovM represses urease expression by directly binding to the urease promoter.

### CsrA Directly Binds to the SD Sequence of the mRNA Encoding the Urease

**FIGURE 4:**
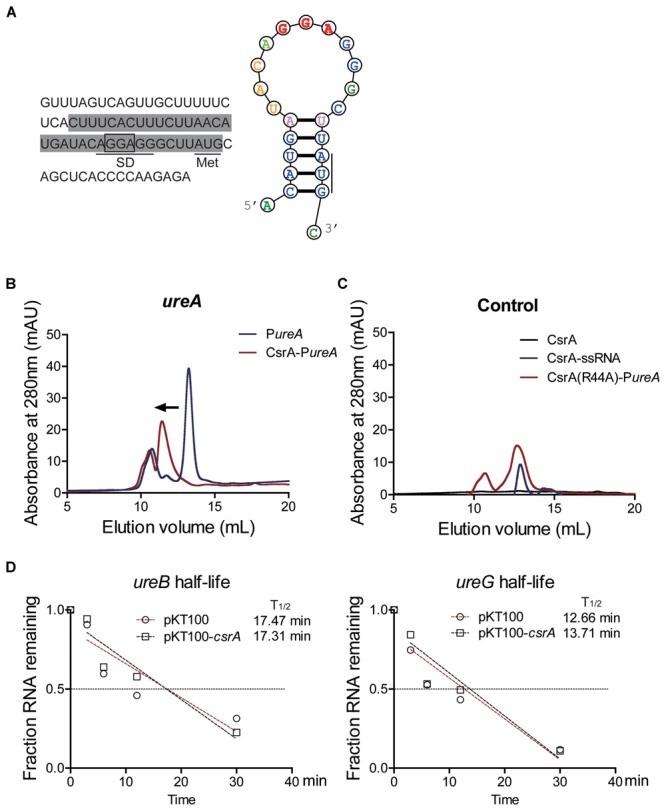
CsrA directly binds to the SD sequence of the mRNA encoding the urease. **(A)** Left: Nucleotide sequences of a portion of the *urease* mRNA 5′UTR. The predicted CsrA binding site is boxed. SD denotes the Shine-Dalgarno sequence of the mRNA encoding the urease. The sequences of synthetic RNA oligonucleotides used for analytical SEC were indicated by shading (5′-CUUUCACUUUCUUAACAUGAUACAGGAGGGCUUAUG-3′, 36 bp). Right: A predicted secondary structure of the postulated urease SD sequence (http://rna.urmc.rochester.edu/RNAstructureWeb/index.html). The GGA binding motif locates in the loop and is highlighted in red bold character. Start codon (AUG) is also lined. **(B)** Analytical SEC of CsrA binding to a predicted urease RNA target. Short RNA oligonucleotides were synthesized. The sequences of synthetic RNA oligonucleotides were derived from the SD sequence of the mRNA encoding the urease and contained the predicted CsrA binding site (illustrated in **A**). The 50 μM CsrA protein and 25 mM RNA samples were used in 50 mM NaCl, 25 mM potassium phosphate buffer, pH = 7.0. The elution was monitored at the absorbance of 280 nm. Binding interactions between CsrA and RNA determined by analytical SEC showing a shift in retention time of the band for unbound RNAs (blue) to faster elution for the complexes (red). **(C)** Same experiment as in **(B)** but using the substituted protein CsrA(R44A). Most of the RNA remains unbound in the presence of the CsrA(R44A) protein (red), indicating a substantially weaker interaction. CsrA protein alone shown in black. ssRNA is a single-stranded RNA with random sequences, which was used as a negative control (5′-CGUCUUGCUAGUGCCGACUAGCGAGAUACACUGAUC-3′, 36 bp). **(D)** RNA half-life assays of *ureB* and *ureG* RNA extracted from *Yptb* wild type (pKT100) and *csrA* overexpression (pKT100-*csrA*) strain. Bacteria were cultured at 26°C to the late exponential phase using a time gradient (0, 3, 6, 12, and 30 min) and rifampicin was added to fix the RNA, then bacteria were harvested and RNA was extracted for qRT-PCR analysis. The level of *ureB* and *ureG* mRNA was normalized to the 16S rRNA level (Circles: pKT100; Squares: pKT100-*csrA*). The gene expression level at 0 min was set as 1. A Linear Regression was performed to determine the relation between Fraction RNA remaining (*Y*-axis) and time (*X*-axis). The sloped dash line in each panel represents the Linear Regression result (Red dashed line: pKT100; Black dashed line: pKT100-*csrA*). In the wild type (pKT100) strain, the time to have half of the initial RNA amount (T_1/2_) for *ureB* and *ureG* mRNA is 17.47 min and 12.66 min, respectively. In the pKT100-*csrA* strain, T_1/2_ for *ureB* and *ureG* mRNA is 17.31 min and 13.71 min, respectively (y represents the Fraction RNA remaining and x represents the Time). Data shown are the averages from four independent experiments.

CsrA is an important component of the carbon storage regulator (Csr) system, which is essential in microorganisms that regulates multiple physiological processes and influences the synthesis of a number of secondary metabolites. Previous research has demonstrated that RovM is regulated by the Csr system (Heroven et al., 2008). Therefore, we investigated whether the urease expression was also regulated by the Csr system, in particular by CsrA. CsrA is an RNA-binding protein that could bind to the SD sequence to block ribosome binding, thus preventing the translation of target mRNA (Dubey et al., 2003). Therefore, to detect the interaction between CsrA and the mRNA encoding the urease (Ypk_1131), we performed analytical SEC to detect stable protein-RNA complexes (Kulkarni et al., 2014). By following the sequence characteristics described in the previous research (Kulkarni et al., 2014) (Supplementary Figure S3), a putative CsrA binding site was predicted. This site overlaps the SD sequence of the mRNA encoding the urease (**Figure 4A**). To study the interaction between CsrA protein and the mRNA encoding the urease, a short RNA was synthesized. The sequences of synthetic RNA oligonucleotides were derived from the SD sequence of the mRNA encoding the urease and contained the predicted CsrA binding sites (the conserved sequence GGA situated in the stem-loop structure) (**Figure 4A**). The 25 μM RNA and 50 μM CsrA proteins were used in the reaction for analytical SEC and absorbance was monitored at 280 nm. As shown in **Figure 4B**, RNA-protein complexes exhibited a considerable shift compared to RNA fragments alone. This result strongly indicated that CsrA has direct interaction with this synthetic RNA. Previous studies showed the arginine at position 44 of CsrA is essential for the binding of CsrA with target RNA (Heeb et al., 2006; Kulkarni et al., 2014). To further confirm the binding of CsrA with target RNA, a point-mutated (R44A) CsrA protein was created and tested for its ability to bind the same SD sequence (Heeb et al., 2006; Kulkarni et al., 2014). The result showed the RNA remaining largely unbound under the same conditions used for the wild type CsrA protein, indicating the point-mutated CsrA protein was unable to interact with the small synthetic RNA (**Figure 4C**). Thus, we observed the specific binding of CsrA with the SD sequence of the mRNA, which suggests CsrA may sequester ribosome binding sites to regulate RNA translation.

Next, we performed RNA half-life experiments to investigate the effect of CsrA on *urease* mRNA stability. To further study the role of CsrA in regulating urease expression, we tried to generate a Δ*csrA* mutant. Unfortunately, we failed to obtain this strain and this may be explained by the fact that the Δ*csrA* mutant was severely affected for growth (Heroven et al., 2008). Therefore, the overexpression approach was used to study the effect of CsrA on urease activity. As shown in **Figure 4C**, the RNA half-life of *ureB* and *ureG* mRNA was not affected by *csrA* overexpression (*ureB*: 17.47 min vs. 17.31 min; *ureG*: 12.66 min vs. 13.71 min) (**Figure 4D** and Supplementary Figure S4). This indicates that CsrA did not affect the degradation of *ureB* and *ureG* mRNA, but may prevent the translation of the mRNA by binding to the SD sequence, thus blocking ribosome binding. Based on these findings, we concluded that CsrA binds to the SD sequence of the mRNA encoding the urease and this may prevent the *urease* mRNA translation.

### CsrA Negatively Regulates Urease Activity

**FIGURE 5:**
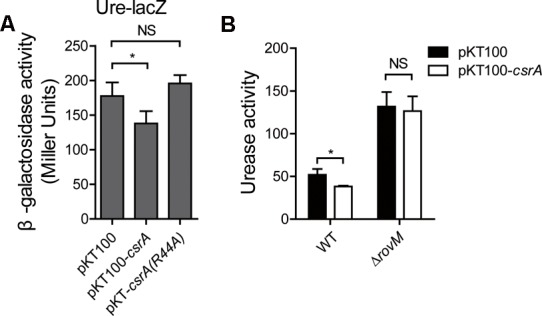
CsrA negatively regulates urease expression. **(A)** β-galactosidase assays for the *Yersinia Pseudotuberculosis* (*Yptb*) wild type (pKT100), *csrA* overexpressing (pKT100-*csrA*) and *csrA* point-mutant (R44A) [pKT100-*csrA(R44A)*] strains. **(B)** Quantitative urease activity assays for the *Yptb* wild type or Δ*rovM* strain overexpressing empty vector (pKT100) or *csrA* (pKT100-*csrA*) in the M9 medium. Urease activity is expressed as micromoles of ammonia produced per minute per milligram of protein. Data shown are the averages and SDs (standard deviations) from at least three independent experiments. ^∗^*p* < 0.05; NS, not significant.

In the transcriptional chromosomal *P_ureABC_ :: lacZ* fusion reporter system, the *ureABC-lacZ* mRNA was transcribed as one fragment which contains the SD sequence of *urease* mRNA. Thus, we tested whether CsrA binds to the SD sequence of *ureABC-lacZ* mRNA to block its translation. If the binding happened, the translation will be blocked and the β-galactosidase activity will be decreased correspondingly. As shown in **Figure 5A**, the overexpression of *csrA* (pKT100-*csrA*) reduced the β-galactosidase activity compared to that in wild type (pKT100), while the overexpression of R44A-mutated *csrA* [pKT100-*csrA(R44A)*] had no effect. These results further support the notion that CsrA blocks *urease* mRNA translation by binding to the SD sequence of the mRNA. Next, we overexpressed *csrA* genes in the wild type and in Δ*rovM* mutant. Both of them were cultured in M9 medium till the stationary phase. It is clear that the urease activity was significantly decreased after CsrA overexpression in the wild type strain. Together with the above SEC results, we have demonstrated that CsrA negatively regulates urease activity through the binding with the SD sequence of the *urease* mRNA.

### Relative Regulatory Effects of OmpR and RovM on Urease Activity

**FIGURE 6:**
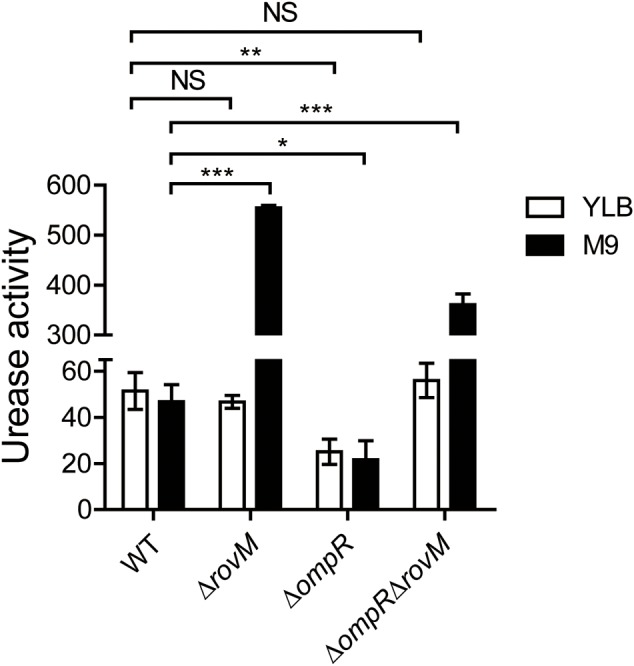
Roles of OmpR and RovM in urease regulation. Quantitative urease activity assays for the *Yersinia Pseudotuberculosis* (*Yptb*) wild type (WT), Δ*ompR*, Δ*rovM* and Δ*ompR*Δ*rovM* strains in M9 or YLB medium. Urease activity is expressed as micromoles of ammonia produced per minute per milligram of protein. Data shown are the averages and SDs (standard deviations) from at least three independent experiments. ^∗∗∗^*p* < 0.001; ^∗∗^*p* < 0.01; ^∗^*p* < 0.05; NS, not significant.

OmpR is the regulator of the two-component system EnvZ/OmpR and it plays an important role in the physiology and virulence of *Y. enterocolitica* (Brzostek et al., 2003). Meanwhile, OmpR was also reported to regulate the acid tolerance response in *Yptb* (Flamez et al., 2008; Zhang et al., 2013). OmpR also has been reported to directly bind to the urease promoter region to activate urease expression in *Yptb* (Hu et al., 2009). To investigate the role of OmpR in regulating urease activity in *Yptb* and the relation between OmpR and RovM, we constructed an OmpR (Δ*ompR*) (Zhang et al., 2013) mutant and an OmpR/RovM double mutant (Δ*ompR*Δ*rovM*). The Δ*ompR* strain had a decreased urease activity compared to that in wild type strain (**Figure 6**). We have already proven that RovM is a negative regulator of urease (**Figure 2**), however we found the urease activity in M9 medium is significantly increased in the Δ*ompR*Δ*rovM* double mutant strain compared to that in the wild type strain (**Figure 6**), suggesting RovM may play a predominant role in regulating urease expression in nutrient-limited condition. RovM has been reported in response to the availability of nutrients and can only be significantly induced in minimal medium (Heroven and Dersch, 2006; Song et al., 2015). Therefore, we also measured the urease activity of these strains in nutrient-rich medium YLB. As shown in **Figure 6**, in nutrient-rich conditions the Δ*rovM* mutant has a normal urease activity. Given the fact that RovM level is very low in complex medium (Heroven and Dersch, 2006), it is reasonable to expect that in nutrient-rich condition (YLB) the regulatory ability of RovM is rather minor and the deletion of this gene did not greatly influence the urease expression. In nutrient-rich conditions, the Δ*ompR* mutant has a reduced urease activity. However, the double mutant stain has a normal urease activity compared to that in the wild type strain. Compared with the positive regulator OmpR (Hu et al., 2009), we demonstrated that the negative regulator RovM plays a dominant role in regulating urease activity.

## Discussion

The acid resistance systems play important roles in the survival of *Yptb* under acid stress and several mechanisms have been reported to strictly regulate their expression (Song et al., 2015). The urease system is an important acid resistance system that can be regulated by many factors. Nutrient conditions are essential for the survival of bacterial, thus we first tested the impact of different nutrient level for urease activity. We clearly showed that urease activity responds to different nutrient conditions, exhibiting high activity in the rich medium while maintaining low activity in poor medium (**Figure 1**). As expected, urease expression in the rich medium increased dramatically and this effect was more significant in acidic conditions (**Figure 1D**). The opposite results were found in minimal medium, as urease was not activated by low pH (**Figure 1D**). A global regulator RovM, had been shown to regulate many acid resistance systems including AR3- and T6SS4-dependent acid survival systems (Song et al., 2015). Many homologs of RovM in different bacteria had been reported. For instance, HexA from *Dickeya dadantii* (formerly known as *Erwinia carotovora*), PecT from *Er*. *chrysanthemi* and LrhA from *E. coli* (Surgey et al., 1996; Mukherjee et al., 2000; Lehnen et al., 2002). These genes participate in different cellular pathways like biofilm formation, virulence and many metabolic activities. However, whether this global regulator RovM could regulate the urease acid resistance system in *Yptb* was still unknown. In this study, we convincingly showed that RovM strongly represses urease activity (**Figure 2**). In addition, as shown in **Figure 2D**, the Δ*rovM* mutant had a significantly higher survival rate under acid stress. This highlighted the importance of urease acid resistance systems in *Yptb* under acid stress. Next, we tried to reveal how RovM regulates urease expression and characterized a RovM binding site in the promoter region of the urease gene (**Figure 3**). Taken together, we demonstrated that RovM represses urease activity and the underlying mechanism is that RovM can directly bind to the urease promoter.

The Csr system functions upstream of RovM and regulates RovM expression (Heroven et al., 2008). Therefore, in the following study we investigated the role of CsrA in urease expression. We first showed that CsrA directly binds to the SD sequence of the mRNA encoding the urease. Interestingly, a mutation of the RNA binding site (R44A) of CsrA abolished this binding (**Figure 4B**), indicating that CsrA could block the SD sequence of the *urease* mRNA and thus regulate urease activity. To further study the role of CsrA in regulating urease expression, we tried to generate the Δ*csrA* mutant. But we were unsuccessful as the Δ*csrA* mutant had a severe growth defect phenotype, as previously reported (Heroven et al., 2008). Nevertheless, we observed that the overexpression of CsrA inhibited the urease activity (**Figure 5B**). Interestingly, the urease activity was significantly elevated in the Δ*rovM* mutant and the urease repression ability of CsrA was abolished in the Δ*rovM* mutant (**Figure 5B**). This suggests the repression ability of CsrA was relatively minor compared to RovM. Meanwhile, CsrA was also reported to up-regulate *rovM* expression (Heroven et al., 2008) and RovM was identified suppress urease expression (**Figures 2, 3**). This indicates CsrA may also inhibit urease activity through the up-regulation RovM. However, further study about the relation between CsrA and RovM was hindered due to the lack of a Δ*csrA* mutant.

Another important two-component regulator, OmpR has been reported to directly bind to the urease promoter region and significantly activates urease expression in *Yptb* (Hu et al., 2009). It plays an important role in acid response since the deletion of OmpR decreased the acid survival of *Yptb* (Hu et al., 2009). Given the opposite regulatory effects of OmpR and RovM on urease expression, we tried to elucidate their relationship in the control of urease activity. Compared with the OmpR-dependent activation, the negative regulator RovM plays a dominant role in regulating urease activity (**Figure 6**). These two factors respond to different stimuli and exhibit different regulatory patterns (Hu et al., 2009; Zhang et al., 2013; Song et al., 2015). Nevertheless, the influence of RovM on urease is clearly different as a function of the nutrient status of the medium (see **Figure 6**). Collectively, in this study we revealed a novel mechanism of urease regulation via RovM that depends on nutrient conditions.

As an acid resistance system in bacteria, urease plays an important role in mitigating acid stress. Several systems have been identified to increase survival under acid conditions. The classical types of acid resistance (AR) systems usually respond to severe acid stress. Apart from the urease system, the aspartate-dependent acid survival system (Hu et al., 2010) and the T6SS (Zhang et al., 2013) also respond to acid stress, significantly increasing survival at pH ≥ 4.5. In *Yptb*, the optimal pH for urease activity is approximately 4.5 (data not shown). Under nutrient-rich conditions, urease was highly expressed. One of the reasons is that the medium provides abundant substrate, and thus urease can hydrolyze urea instantly allowing rapid bacterial growth and helping the strain proliferate efficiently. Meanwhile, the up-regulation of urease in nutrient-rich conditions increases the virulence of bacterial pathogens (Rutherford, 2014). Thus, many enteropathogens express a high level of urease to exert strong pathogenicity in the alimentary canal, since this place contains rich resources. Under nutrient-limited conditions, urease is dramatically repressed by multiple pathways. Because of the shortage of nutrients, there is inadequate substrate available for urease metabolism, and a stringent response is initiated, thereby preventing urease expression and saving limited energy and nutrition for maintaining fundamental metabolic processes. We propose that to ensure long-term survival, with the nutrient limitation like sub-optimal conditions within the host, bacteria will decrease their virulence and deliver fewer toxins to the host.

**FIGURE 7:**
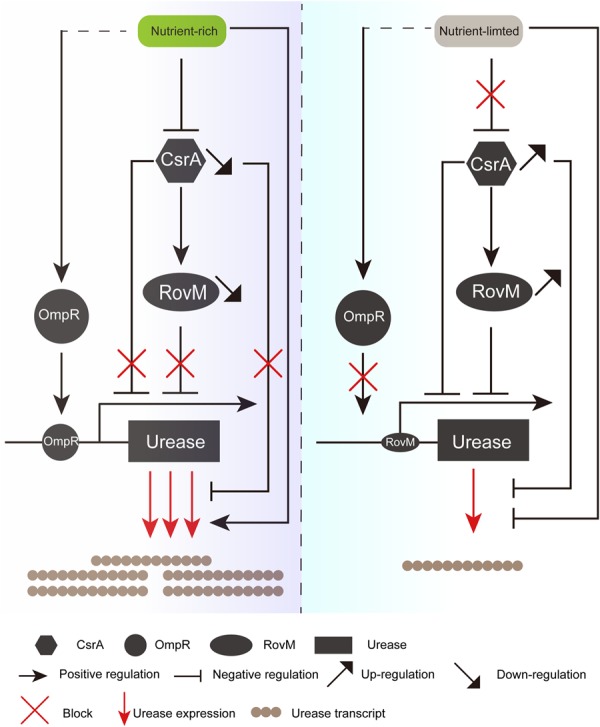
Models for the regulation of urease by RovM in response to nutrient conditions. In nutrient-rich conditions, CsrA is sequestered and RovM is down-regulated, causing urease to be released from repression by CsrA and RovM. Meanwhile, urease expression is activated by OmpR (left). In contrast, under nutrient-limited conditions, CsrA is highly expressed and RovM is up-regulated. Thus, the urease expression is down-regulated at both the transcriptional and post-transcriptional levels. Meanwhile, urease expression cannot be activated by OmpR (right).

In summary, we propose a model in which urease is regulated in response to the availability of nutrients (**Figure 7**). When resources for bacterial growth are abundant, CsrA is sequestered and the major regulator RovM is down-regulated by nutrients. In addition, the sequestered CsrA is not able to increase RovM level (Heroven and Dersch, 2006) (**Figure 7**). Nutrients are the predominant factors that increase urease activity. With rich nutrient conditions, RovM and CsrA are suppressed while the urease activator OmpR is activated, leading to high urease expression. On the other hand, when nutrients are limited, CsrA is up-regulated and RovM is activated while the OmpR is deactivated. Therefore, the urease is down-regulated at both the transcriptional and post-transcriptional levels. If nutrients are insufficient in the environment, urease is repressed rigorously through the sequential CsrA-RovM pathway. We believe that it is still necessary to elucidate the mechanism by which the Csr system regulates urease, as the Csr system is involved in a large and complex network that is common among bacteria. The Csr genes contain a large number of factors that participate in diverse regulatory pathways within the upstream and downstream regions. Meanwhile, CsrB and CsrC antagonism of CsrA, as well as that between CsrB and CsrC, have complex effects (Heroven et al., 2012). These have made it difficult to fully explain the detailed functions of the Csr system and continuing work is required.

## Conclusion

In this study, we revealed that acid resistance urease expression is influenced by different nutrient conditions. RovM represses urease expression by directly binding to the urease promoter and CsrA down-regulates urease activity possibly by its binding to the SD sequence of the mRNA encoding the urease. This study will shed new light on the understanding of *Yptb* acid resistance mechanisms and provide more possibilities to control bacterial infection.

## Author Contributions

QD, LXu, XS, and YW designed the research. QD, LXu, LXi, KZ, YS, CL, and LZ performed the research. QD, LXu, XS, and YW analyzed the data. XS and YW contributed the new reagents and analytic tools. QD, LXu, XS, and YW wrote the paper.

## Conflict of Interest Statement

The authors declare that the research was conducted in the absence of any commercial or financial relationships that could be construed as a potential conflict of interest.
