# Supplementary material for: RovM and CsrA Negatively Regulate Urease Expression in Yersinia pseudotuberculosis
Source: Front Microbiol. 2018 Feb 27;9:348. doi: 10.3389/fmicb.2018.00348 (PMC5835112; doi:10.3389/fmicb.2018.00348)
Supplement: Supplementary file 1 [file Data_Sheet_1.pdf]

Supplementary Materials for

RovM and CsrA Negatively Regulate Urease Expression in *Yersinia pseudotuberculosis*

Qingyun Dai[1#], Lei Xu[2, 3#], Lu Xiao[1], Kaixiang Zhu[1], Yunhong Song[2], Changfu Li[1], Lingfang Zhu[2], Xihui Shen[2, 3]*, and Yao Wang[1]*

[1] State Key Laboratory of Crop Stress Biology for Arid Areas and College of Life Sciences, Northwest A&F University, Yangling, Shaanxi 712100, China.

[2] College of Life Sciences, Northwest A&F University, Yangling, Shaanxi 712100, China.

[3] Shaanxi Key Laboratory of Agricultural and Environmental Microbiology, College of Life Sciences, Northwest A & F University, Yangling, Shaanxi 712100, China

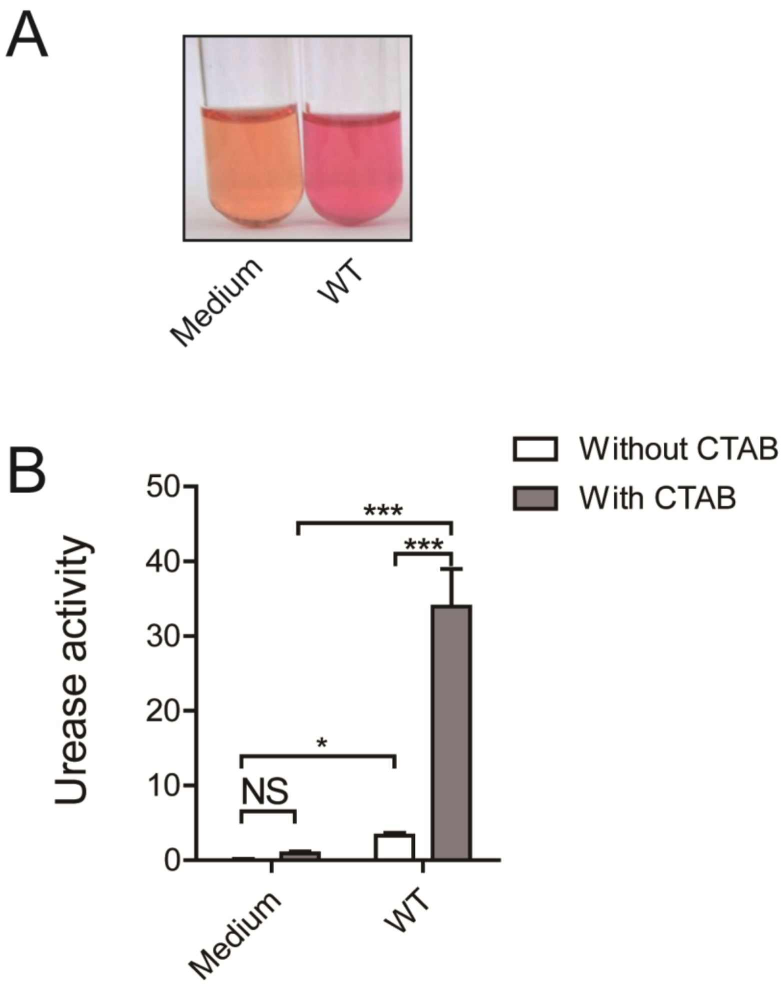

Figure S1. Qualitative and quantitative assays of urease activity.

A. Qualitative assays of urease activity in YLB medium with or without *Yersinia pseudotuberculosis* (*Yptb*) wild type (WT). pH was indicated by phenol red.
B. Quantitative assays of urease activity in YLB medium with or without *Yersinia pseudotuberculosis* (*Yptb*) wild type (WT). The quantitative test buffers with [0.1 % (w/v) cetyldimethylammonium bromide (CTAB), 0.6 % (w/v) NaCl, 100mM Citrate, 5 mM urea, pH = 6.0] or without CTAB [0.6 % (w/v) NaCl, 100mM Citrate, 5 mM urea, pH = 6.0] were used. The medium that without CTAB was served as negative control and the urease activity of this group was set as 0. Urease activity is expressed as micromoles of ammonia produced per minute per milligram of protein. Data shown are the averages and SDs (standard deviations) from at least three independent experiments. ***p < 0.001, *p < 0.05, NS, not significant.

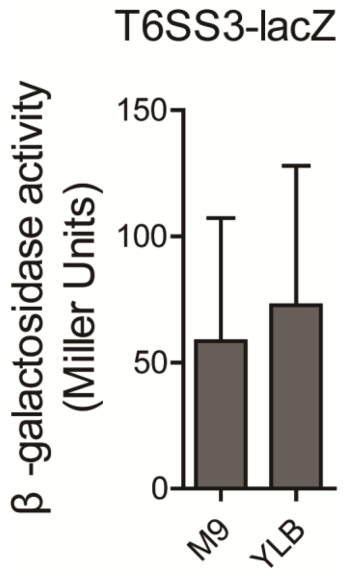

Figure S2. Different culture medium does not affect T6SS3 promoter related β-galactosidase activity.

β-galactosidase assays of T6SS3 promoter activity of the *Yptb* wild type (WT) strains cultured in YLB and M9 medium. Data shown are the averages and SDs (standard deviations) from at least three independent experiments.

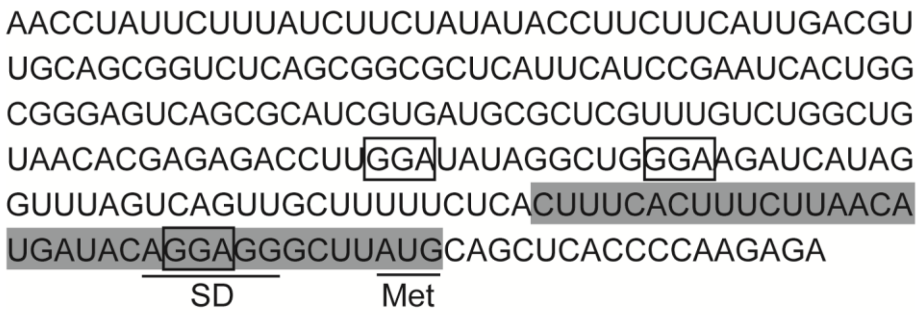

Figure S3. Identification of the CsrA binding sites.

Nucleotide sequences of a portion of the *urease* mRNA 5’UTR. Three predicted CsrA binding sites are boxed. SD denotes the Shine-Dalgarno sequence of the mRNA encoding the urease. The sequences of synthetic RNA oligonucleotides used for analytical size exclusion chromatography (SEC) were indicated by shading (5’-CUUUCACUUUCUUAACAUGAUACAGGAGGGCUUAUG-3’, 36bp).

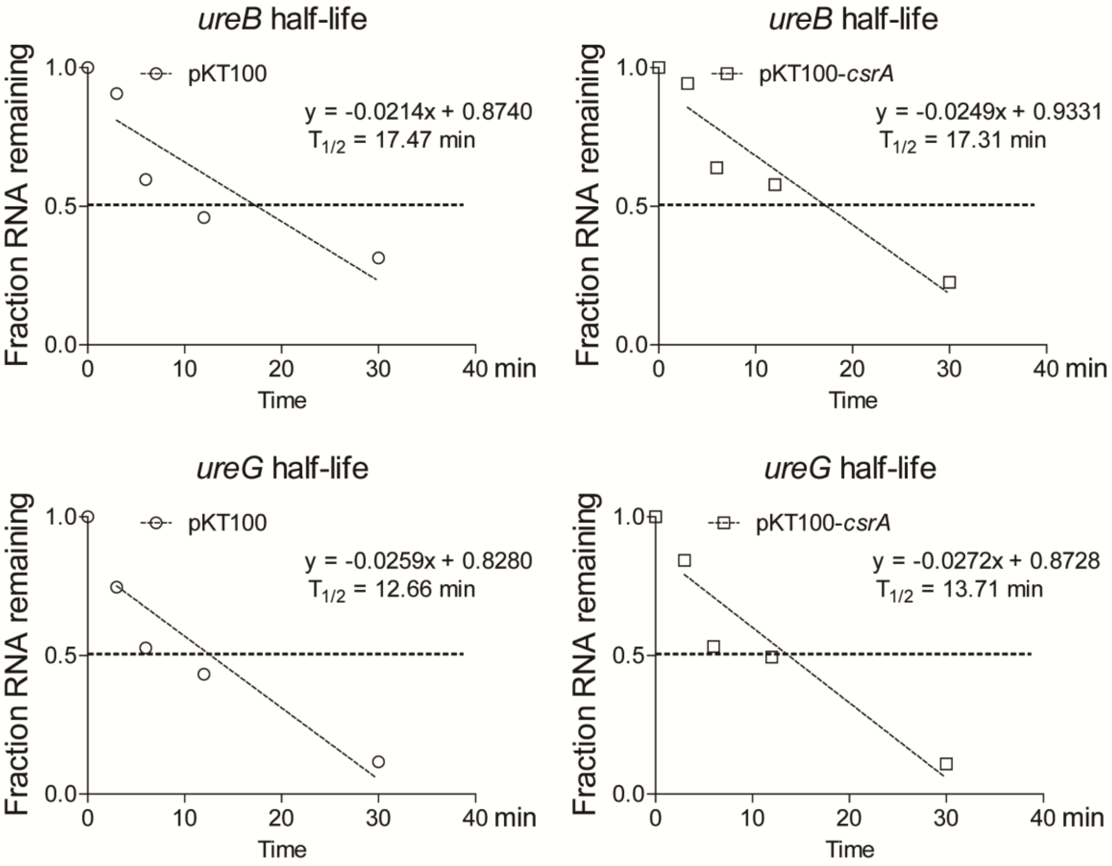

Figure S4. RNA half-life assays of *ureB* and *ureG* gene.

RNA half-life assays of *ureB* and *ureG* RNA extracted from *Yptb* wild type (pKT100) and *csrA* overexpression (pKT100-*csrA*) strain. Bacteria were cultured at 26 ℃ to the late exponential phase using a time gradient (0 min, 3 min, 6 min, 12 min and 30 min) and rifampicin was added to fix the RNA, then bacteria were harvested and RNA was extracted for qRT-PCR analysis. The level of *ureB* and *ureG* mRNA was normalized to the 16S rRNA level (Circles: pKT100; Squares: pKT100-*csrA*). The gene expression level at 0 min was set as 1. A Linear Regression was performed to determine the relation between Fraction RNA remaining (Y-axis) and time (X-axis). The sloped dash line in each panel represents the Linear Regression result (Red dashed line: pKT100; Black dashed line: pKT100-*csrA*). In the wild type (pKT100) strain, the time to have half of the initial RNA amount ($T_{1/2}$) for *ureB* and *ureG* mRNA is 17.47 min (y = -0.0214x + 0.874) and 12.66 min (y = -0.0259x + 0.828), respectively. In the pKT100-*csrA* strain, $T_{1/2}$ for *ureB* and *ureG* mRNA is 17.31 min (y = -0.0249x + 0.9311) and 13.71 min (y = -0.0272x + 0.8728), respectively (y represents the Fraction RNA remaining and x represents the Time). Data shown are the averages from four independent experiments.

Supplement1. The strains and plasmids used in this study

Strain or plasmid	Relevant characteristics	Reference
E. coli		
S17-1λ *pir*	*λ-pir* lysogen of S17-1, *thi pro hsdR hsdM*$^+$ *recA* RP4 2-Tc::Mu-Km::Tn7	Simon *et al.*, 1983
Y. pseudotuberculosis		
YPIII	Wild-Type *Y. pseudotuberculosis*, Nal^r	Rosqvist *et al.*, 1988
Δ*ompR*	*ompR* gene deleted in YPIII, Nal^r	Zhang *et al.*, 2013
Δ*rovM*	*rovM* gene deleted in YPIII, Nal^r	This study
Δ*ureC*	*ureC* gene deleted in YPIII, Nal^r	This study
Δ*rovM*Δ*ureC*	*rovM* and *ureC* genes deleted in YPIII, Nal^r	This study
WT(pKT100)	YPIII containing pKT100, Nal^r, Km^r	Zhang *et al.*, 2013
Δ*rovM*(pKT100)	Δ*rovM* containing pKT100, Nal^r, Km^r	This study
Δ*rovM*(pKT100-*rovM*)	Δ*rovM* containing pKT100-*rovM*, Nal^r, Km^r	This study
Δ*rovM*(pKT100-*CsrA*)	Δ*rovM* containing pKT100-*CsrA*, Nal^r, Km^r	This study
WT(pKT100-*CsrA*)	YPIII containing pKT100-*CsrA*, Nal^r, Km^r	Zhang *et al.*, 2013
WT(pKT100-*CsrA* R44A)	YPIII containing pKT100-*csrA*(R44A), Nal^r, Km^r	This study
**Plasmid**		
pKT100	Cloning vector, p15A replicon, Km^r	Hu *et al.*, 2009
pKT100-*rovM*	*rovM* under control of its own promoter in plasmid pKT100	This study
pKT100-*csrA*	*csrA* under control of its own promoter in plasmid pKT100	This study
pKT100-*csrA(R44A)*	Argine in the site of 44 mutant into Alanine of *csrA* under control of its own	This study

	promoter in plasmid pKT100	
pDM4	Suicide vector, *mob*RK2, *ori*R6K, *pir*, *sacB*, Cm^r	Milton *et al.*, 1996
pDM4-Δ*ureC*	Construct used for in-frame deletion of *ureC*, Cm^r	This study

*Nal^r, Km^r, Amp^r and Cm^r represent resistance to nalidixic, kanamycin, ampicillin and chloramphenicol, respectively.

Supplement2. The primes used in this study

Primer	Sequence	Note
Ypk_1133M1F Sal I	ACGCGTCGACGAGATGGCAAATCCGTAG	*ureC* upstream, to generate pDM4-Δ*ureC*
Ypk_1133M1R	TAGACCCGCGTATTCTT	
Ypk_1133M2F	AAGAATACGCGGGTCTAGCCACCTGTGAGCCAATT	*ureC* downstream, to generate pDM4-Δ*ureC*
Ypk_1133M2R BglI I	GGAAGATCTTTCATCCGGTGAACGACT	
rovM-1F-Sal I	CTCGGTCGACTGTGGGCTAGATCCATCC	*rovM* upstream, to generate pDM4-Δ*rovM*
rovM-1R	AGCAGCAGCAGCAGCAAA	
rovM-2F	TTTGCTGCTGCTGCTGCTGGGTTGCCTGGTTTACCT	*rovM* downstream, to generate pDM4-Δ*rovM*
rovM-2R-Bgl II	CTCGAGATCTCTGTGGGCTTTTTACTCC	
rovMF-BamH I	CTGCGGATCCTTTATCCCTATTCATTCTCG	To generate pKT100-*rovM* also to generate pET28a-*rovM*
rovMR-Sal I	CTGCGTCGACTTAATCTTCATCACCTGTC	
csrA-F-BamH I	GCGCGGATCCGGACAATGGTCGATGAC	To generate pKT100-*csrA* also to generate pET28a-*csrA*,
csrA-R-Sal I	GGGCGTCGACGTTACACGAGACGCTGC	
ureABCp1000F-Sal I	ACGCGTCGACCTGCCTGTAATTTATTGGCGTC	To generate pDM4-P_{ureABC} ::

ureABCpR-Xbal I	CTAG<u>TCTAGA</u>GGTGAGCTGCATAAGCCCTC	*lacZ*
UreB-F<br>UreB-R	CGAAAGACAGTAAAGAACAGAA<br>GAAGAAATGGAAATGGGAC	qRT-PCR Primer for *ureB*
UreE-F<br>UreE-R	TGTATGTGCCTCTGACCG<br>CACCACCAAACAGTAGCC	qRT-PCR Primer for *ureE*
UreG-F<br>UreG-R	AGGATGCCAAACAGGTAA<br>AATCAGGTCGCTTTCAGG	qRT-PCR Primer for *ureG*
RovM-F<br>RovM-R	GGTAAAGCGCAGCCCATT<br>AAAGGGACTGGCTCTCCG	qRT-PCR Primer for *rovM*
16S rRNA-F<br>16S rRNA-R	CTAGCGATTCCGACTTCAT<br>CCCTTATCCTTTGTTGCC	qRT-PCR Primer for 16s rRNA
ureABC-F<br>ureABC-R	GATTGGATGCTTTTTAATTTATTG<br>GCAAGCTAAAATCAAGACAAATTA	The probe for EMSA
ureC-NC-F<br>ureC-NC-R	CATCTCATCTCCCCGCAAC<br>ACCTTTCCCCAAAATACCAAC	The probe for EMSA, negative control
P*ure*-CsrA RNA	CUUUCACUUUCUUAACAUGAUACAGGAGGGCUUAUG	for SEC,RNA primer
csrA-R44A M1R<br>csrA-R44A M2F	GATTTCTTCAGCGTGAAC<br>GTTCACGCTGAAGAAATC	For *csrA* point-mutated (R44A)
csrAexF BamH I<br>csrAexR Sal I	CGC<u>GGATCC</u>ATGCTTATTCTGACTCGTCG<br>ACGC<u>GTCGAC</u>TCAGTAAGTCGTCGGTTGAG	For pET28a-csrA (R44A)

*Underlined sites indicate restriction enzyme cutting sites added for cloning.

References:

Simon, R., Priefer, U., and Pühler, A. (1983). A Broad Host Range Mobilization System for In Vivo Genetic Engineering: Transposon Mutagenesis in Gram Negative Bacteria. *Bio/Technology* 1**,** 784. doi: 10.1038/nbt1183-784.

Rosqvist, R., Skurnik, M., and Wolf-Watz, H. (1988). Increased virulence of Yersinia pseudotuberculosis by two independent mutations. *Nature* 334**,** 522-524. doi: 10.1038/334522a0.

Zhang, W., Wang, Y., Song, Y., Wang, T., Xu, S., Peng, Z., Lin, X., Zhang, L., and Shen, X. (2013). A type VI secretion system regulated by OmpR in Yersinia pseudotuberculosis functions to maintain intracellular pH homeostasis. *Environ Microbiol* 15**,** 557-569. doi: 10.1111/1462-2920.12005.

Milton, D.L., O'toole, R., Horstedt, P., and Wolf-Watz, H. (1996). Flagellin A is essential for the virulence of Vibrio anguillarum. *J Bacteriol* 178**,** 1310-1319.
